# Using HEK293T Expression System to Study Photoactive Plant Cryptochromes

**DOI:** 10.3389/fpls.2016.00940

**Published:** 2016-06-27

**Authors:** Liang Yang, Xu Wang, Weixian Deng, Weiliang Mo, Jie Gao, Qing Liu, Chuanyu Zhang, Qin Wang, Chentao Lin, Zecheng Zuo

**Affiliations:** ^1^Laboratory of Soil and Plant Molecular Genetics, College of Plant Science, Jilin UniversityChangchun, China; ^2^Basic Forestry and Proteomics Research Center, Haixia Institute of Science and Technology, Fujian Agriculture and Forestry UniversityFuzhou, China; ^3^Department of Molecular, Cell and Developmental Biology, University of California, Los AngelesCA, USA

**Keywords:** cryptochrome, expression system, linear DNA, photo-biochemical activity, HEK293T

## Abstract

Cryptochromes are photolyase-like blue light receptors that are conserved in plants and animals. Although the light-dependent catalytic mechanism of photolyase is well studied, the photochemical mechanism of cryptochromes remains largely unknown. Lack of an appropriate protein expression system to obtain photochemically active cryptochrome holoproteins is a technical obstacle for the study of plant cryptochromes. We report here an easy-to-use method to express and study *Arabidopsis* cryptochrome in HEK293T cells. Our results indicate that *Arabidopsis* cryptochromes expressed in HEK293T are photochemically active. We envision a broad use of this method in the functional investigation of plant proteins, especially in the large-scale analyses of photochemical activities of cryptochromes such as blue light-dependent protein–protein interactions.

## Introduction

Plants possess several photoreceptors mediating light regulation of gene expression and photo-physiologic response (Lin, 2002; Yu et al., 2010). Cryptochromes are photolyase-like blue light receptors identified first in plants (Ahmad and Cashmore, 1993; Cashmore, 2003; Lin and Shalitin, 2003; Sancar, 2003). *Arabidopsis* cryptochrome1 (CRY1) primarily mediates inhibition of hypocotyl elongation while *Arabidopsis* cryptochrome2 (CRY2) regulates photoperiodic floral initiation (Ahmad and Cashmore, 1993; Guo et al., 1998). It has been suggested that cryptochromes undergo blue-light-dependent conformational changes to interact with several signaling proteins and transduce the blue light signal (Partch et al., 2005; Yu et al., 2007). Although cryptochromes and their co-factors have been extensively studied for decades (Liu et al., 2008; Liu B. et al., 2011; Zuo et al., 2011; He et al., 2015), the exact mechanism of cryptochrome initiating and coordinating its interacting-proteins to transduce the signal is still not fully characterized (Liu H. et al., 2011). The lack of efficient expression systems that yield functional holoproteins impedes detailed biochemical studies of cryptochrome-mediated blue light signal transduction. In previous studies, we have expressed and purified cryptochromes and their interacting proteins in various expression systems, e.g., *Escherichia coli*, yeast, and insect cell (Malhotra et al., 1995; Liu et al., 2008; Zuo et al., 2011). However, all those systems have major drawbacks, which limit their use in a wide range of biochemical analyses. These disadvantages include deficient chromophore, toxicity, low yield, and light independent constitutive activities.

The HEK293T cell line was generated more than 30 years ago, which has been extensively used as an expression vehicle for recombinant proteins (Graham et al., 1977). The biochemical machinery of HEK293T cell is suitable for expressing most of mammalian and non-mammalian proteins requiring post-translational modification and protein folding (Thomas and Smart, 2005). However, this expression system has not been widely used in the study of plant proteins. Recently, *Arabidopsis* cryptochrome2 itself has been suggested as an Optogenetics module to activate mammalian signaling pathway under blue light (Bugaj et al., 2013; Konermann et al., 2013; Taslimi et al., 2014). On the other hand, we and several other laboratories have transiently expressed plant genes in HEK293T expression system to study the photo-biochemical mechanism of plant photoreceptors. For example, *Arabidopsis* CRY2 has been shown to form photobody in response to blue light in HEK293T cells (Ozkan-Dagliyan et al., 2013). We have also used HEK293T to study protein–protein interactions of *Arabidopsis* AtCRY1 (Gao et al., 2015). Furthermore, AtCRY1–AtPIF4/5 interaction has been studied in HEK293T expression system (Pedmale et al., 2015). These reports suggest that HEK293T system could be suitable for the expression of photoactive plant proteins. We therefore sought to develop an easy-to-use protocol for optimal expression of photoactive plant proteins in HEK293T cells. Here, we describe a simplified method for transiently expressing plant photoactive protein in HEK293T cells. This method is suitable for 96-well plate format and using a polymerase chain reaction (PCR) product as template for recombinant protein expression. It is expected that this methodology would eventually be applied for large-scale protein–protein interaction identification or biochemical-activity analyses for plant proteins.

## Materials and Methods

### HEK293T Cell Culture

HEK293T (ATCC^®^ CRL-11268^TM^) cells were maintained in DMEM (Thermo, 10569-044) supplemented with 10% (v/v) FBS (Thermo, 10100147), 100 U/ml penicillin, and 100 mg/ml streptomycin (Hyclone, SV30010) in Cell Culture Flask, T75 (Eppendorf, 0030711.122). For subculture, after the cell count reaching 2 × 10^7^, discarded the spent medium and washed the cells with PBS (pH7.2, Thermo, 20012050) gently; Incubated the washed cells with pre-warmed TrypLE^TM^ Express (Nestler et al., 2004) (Thermo, 12605-010) at room temperature for 2 min, shook the flask softly and then observed those cells under the inverted microscope; if more than 90% of cells were detached, added 2 volumes of TrypLE^TM^ Express medium, mixed well and transferred the mixture into a 15 ml Coning tube, then centrifuged at 200 g for 5 min; discarded the supernatant, re-suspended the pellet with 4 ml medium; cells were counted and then transferred into new T75 flask, 6-well plates, 10 cm-dishes or 96-well plates (Shaw et al., 2002; Aricescu et al., 2006).

### Construction of Linear DNA

To generate the linear DNA encoding AtCRY2–AcGFP, the following primers were used: SV 40 F: GCAGCACCATGGCCTGAAA, SV 40 AtCRY2–AcGFP R: TTTGTCCATCTTCATAAGCTTTTTGCAAAAGCCTA, AtCRY2–AcGFP F: ATGAAGATGGACAAAAAGAC, AtCRY2–AcGFP R: TCACTTGTACAGCTCATCCATG. The DNA fragment of SV40 and AtCRY2–AcGFP, both of which contain 15 bp overlap, were generated by PCR, respectively. The PCR product of those two fragments were diluted and mixed in the second round PCR. The linear DNA containing SV40 and AtCRY2–AcGFP was generated by overlap PCR (Liu et al., 1997). For the construction of linear DNA including 4xMyc AtCIB1 and 4xMyc AtSPA1, the same protocol was applied and it performed with the following primers: SV40 AtCIB1R: GTTAATTAACCCCATAAGCTTTTTGCAAAAGCCTA, SV40 AtSPA1 R: TCACCGTTAATTAACCCCATAAGCTTTTTGCAAAA, AtCIB1 F: ATGGGGTTAATTAACGGTGA, AtCIB1 R: TCAAACTCCTAAATTGCCAT, AtSPA1 R: TCAAACAAGTTTTAGTAGCT. PCR products were purified by purification kit (TianGen, DP204).

### Lipofectamine^®^ 2000 Based Transfection

According to the manufacture manual, PCR products were first mixed with 10 μl Opti-MEM^®^ (Thermo, 31985070) in a 96-well plate, before adding Lipofectamine^®^ 2000 Transfection (Thermo, 11668019) at the transfection-reagent/DNA ratio of 3 μl:1 μg. After 5 min incubation, the mixture was mixed with HEK293T gently; cells were then cultured under the condition of 37°C, 5% CO_2_ for 16 h.

### Lipofectamine^®^ 3000-Based Transfection

For Lipofectamine^®^3000 (Thermo, L300015) base transfection, the transfection reagents and DNA were mixed in two steps: (i) Diluted the linear DNA with Opti-MEM, added P3000 at the transfection-reagent/DNA ratio of 2 μl:1 μg, (ii) Diluted the linear DNA with Opti-MEM, added Lipofectamine3000 at the transfection-reagent/DNA ratio of 1.5 μl:1 μg; those two kinds of mixture were then combined at the final volume of 10 μl. After incubation at RT for 5 min, dropped the mixture to the cells. For 6-well or 10 cm-plate format transfection, the transfection-reagent/DNA ratio was kept and the total reaction volume was scaled up accordingly. The cell cultivation was as described above.

### Calcium Phosphate-Based Transfection

0.2 μg linear DNA was diluted with Opti-MEM at the final volume of 5 μl, then added 0.25 μl (20×) 2.5 M CaCl_2_, and 5 μl 2xHeBS (250 mM NaCl, 10 mM KCl, 1.5 mM Na^2^HPO4, 12 mM Dextrose, and 50 mM Hepes). After incubation at RT for 5 min, aspirated media from 96-well plate, slowly dropped the DNA mixture to coat the whole well and then added 100 μl fresh media and 25 μM Chloroquine (Sigma C6628). Cells were incubate in 37°C, 5% CO_2_ incubator for 6 h or overnight and transferred into fresh media without Chloroquine (Batard et al., 2001). For 6-well plates or 10 cm-dishes format, all reagents were applied with the same ratio of 96-well-plate format, except that the total reaction volume was scaled up accordingly.

### Protein Purification and Spectrophotometry

The transfected cells were washed with PBS pH7.2 and detached by TrypLE^TM^ Express (1X). The detached cells were collected by centrifuge at 800 g for 5 min and lysed by lysis buffer (25 mM Tris–HCl pH 7.4, 150 mM NaCl, 1% NP-40, 1 mM EDTA, 5% glycerol) containing EDTA-free Protease Inhibitor Cocktail Tablets (Roche, 4693159001). After incubation on ice for 15 min, the lysate were centrifuged 12000*g* for 10 min. The supernatant was incubated with HisPur^TM^ Ni-NTA Superflow Agarose Beads (Thermo, 25215) at 4°C for 3 h. After wash for 5 times, recombinant AtCRY2 and AtCRY2^D387A^ were eluted by 300 mM imidazole (Sigma I1533). The absorption of recombinant AtCRY2 and AtCRY2^D387A^ were detected by Multiskan^TM^ GO Microplate Spectrophotometer (Thermo, 51119300).

### Microscopy of CRY2–AcGFP, CRY2^D387A^–AcGFP

Cells were cultured by Glass Bottom Cell Culture Dish (NEST, 801001) and transfected with AtCRY2–AcGFP, AtCRY2^D387A^–AcGFP by Lipofectamine^®^ 2000 method. After 24 h, AcGFP was observed with Zeiss observer A1. For calculation of transfection efficiency, cells were re-suspended with PBS (pH 7.2), and cells with or without fluorescence signal were counted on blood counting chamber. GFP and DAPI signals were observed with Zeiss LSM880 (488-nm laser channel and 405-nm laser channel). The figures were merged by Zeiss ZEN 2 software.

### Co-Immunoprecipitation

The HEK293 cells co-transfected with CRY2-CIB1 or CRY2-SPA1 were incubated in dark condition. Cells were irradiated with blue light (50 μmol m^-2^ s^-1^) for indicated time before harvest. After wash with PBS pH 7.2 and detaching by TrypLE^TM^ Express (1X), the detached cells were collected by centrifuge at 800*g* for 5 min and lysed by Pierce IP Lysis Buffer (87787, Pierce) supplemented with EDTA-free Protease Inhibitor Cocktail Tablets. After incubation on ice for 15 min, the lysate was centrifuged 12000*g* for 10 min. The supernatant was incubated with 10 μl AtCRY2 antibody, 20 μl protein A Agrose Beads at 4°C for 1.5 h. Beads were washed 5 times by washing buffer (20 mM HEPES [pH 7.5], 40 mM KCl, and 1 mM EDTA). 30 μl loading buffer was added before immunoblot.

## Results

### Development and Optimization of the PCR-Product Based HEK293T Expression System for Cryptochromes

**FIGURE 1:**
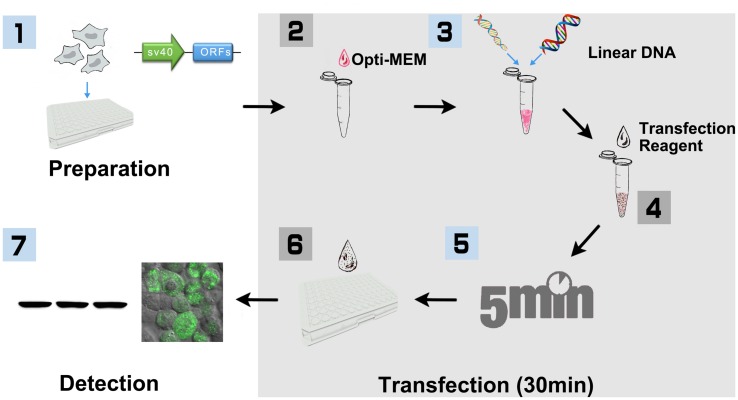
**Workflow of PCR product based HEK293 cell transfection (96-well-plate format with Lipofectamine 2000).** (1) HEK293T cells were cultured in DMEM medium supplemented with 10% FBS and 100 U/ml penicillin, and 100 mg/ml streptomycin under the condition of 37°C, 5% CO_2_. Cells were seeded into 96-well-plate for ∼24 h before transfection. At the same day, SV40 promoter and plant ORF were amplified, respectively, with primers that include 15 bp of overlap sequence. The linear DNA including SV40 promoter and plant ORF were then generated with overlap PCR, purified and quantified for transfection. (2) HEK293T cells were cultured until 80–90% confluence is reached. Added 10 μl Opti-MEM into a sterile tube. (3) Added 0.2 μg linear DNA and vortex. (4) Added 0.6 μl Lipofectamine 2000 and vortex. (5) Incubated at room temperature for 5 min. (6) Added the mixture to 96-well-plate, 10 μl per well, and shook the plate gently. (7) After incubation for 16–32 h, cells were then collected and lysed for Biochemistry assay.

**FIGURE 2:**
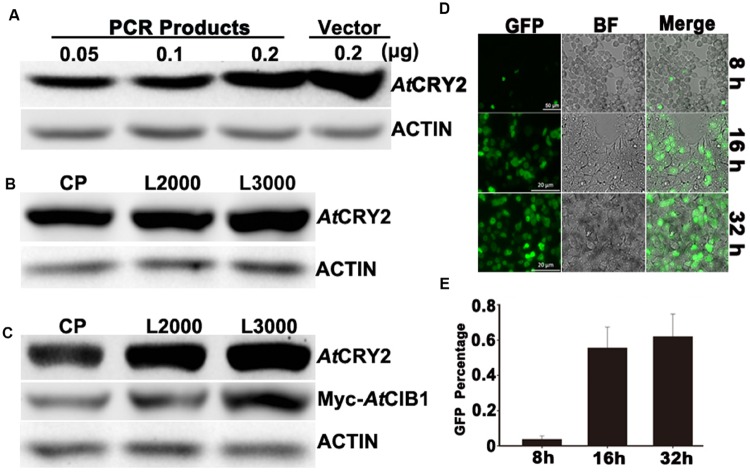
**Optimization of linear-DNA based transfection.** **(A)** Optimization of the amount of linear-DNA based transfection. 0.05 , 0.1, 0.2 linear and 0.2 μg of plasmid DNA encoding AtCRY2 were transfected with the optimized Lipofectamine 2000, respectively. After 24-h incubation, total protein of each sample was probed by the antibody to AtCRY2, stripped and re-probed by the antibody to Actin. **(B)** Efficiency testing of different transfection reagents. 0.2 μl linear DNA encoding AtCRY2 were transfected with Calcium Phosphate, Lipofectamine^®^2000 or Lipofectamine^®^ 3000, the optimized protocols were applied for those transfection reagents, respectively. After transfection and incubation, the immunoblot was performed as described in **(A)**. **(C)** Efficiency testing of co-transfection with different transfection reagents. 0.3 μg linear DNA encoding AtCRY2 and Myc-AtCIB1 were co-transfected with different transfection reagent. The expression level of AtCRY2 and Myc-AtCIB1, transfected with indicated transfection reagent, was shown. The immunoblot was performed as described in **(A)**. **(D)** The microscopy of HEK293T cells transfected with AtCRY2–GFP. Cells were transfected and then incubated with indicated time before microscopy. The GFP and BF (Bright field) channels were merged with Zeiss Zen2 software. **(E)** The efficiency testing of different post-transfection incubation time. After transfection and incubation for indicated time, cells were detached by Recombinant trypsin and re-suspended with PBS (pH 7.2). The efficiency of AtCRY2–GFP was calculated with the following equation: [cell with GFP signal/total counted cell] × 100%.

To develop a PCR-product based and easy-to-use expression system, we first investigated whether the linear-DNA based transfection was efficient enough for plant protein expression in HEK293T cells. The standard non-viral transfection has been routinely performed with a circular plasmid encoding the protein of interest (Son et al., 2000). Although it has been suggested that the plasmids are more efficient than linear-DNA during transfection (Chen and Okayama, 1987), the procedure of HEK293T-based transient transection has been much improved for decades (Son et al., 2000; Tabatt et al., 2004; Chen et al., 2011). As the procedure shown in **Figure 1**, we generated the linear-DNA, which encodes AtCRY2–GFP fusion protein driven by SV40 promoter, by overlap PCR. The transfection reagent, Lipofectamine^®^2000, was selected for optimizing the linear-DNA based transfection. When transfected with different amount of linear-DNA in 96-well-plate, the AtCRY2–GFP expression level increased with increasing linear-DNA amount, and achieved a maximum value at the linear-DNA amount of 0.2 μg per well, which is comparable with the expression level of plasmid-based transfection at plasmid amount of 0.2 μg per well (**Figure 2A**).

We next investigated the efficiencies of linear-DNA-based transfection with different widely used transfection reagents (Calcium Phosphate, Lipofectamine^®^2000 and Lipofectamine^®^ 3000). An equal amount of linear-DNA was used in each transfection assay and only those DNA/transfection-reagent compositions with the highest transfection efficiency were selected, respectively. As shown in **Figure 2B**, the expression level of AtCRY2–GFP was identical with different transfection reagents. Those results suggested that the PCR product based transfection procedure (**Figure 1**) is sufficient for the expression of cryptochrome in HEK293T cells, even with the custom-designed transfection reagent (Calcium Phosphate). At present, there is an increasing requirement for a method on the study of protein functions, which could co-express multiple proteins. We therefore investigated the efficiency of those transfection reagents during linear-DNA-based co-expression assay, which co-transfects AtCRY2–GFP and Myc-CIB1 in HEK293T cells. Our result showed that although the three kinds of transfection reagents exhibited the similar transfection efficiency for single protein expression, the Lipofectamine^®^ 3000 is the most stable for the co-transfection with multiple linear-DNA (**Figure 2C**).

### Optimization of Post-transfection Incubation Time

In the first set of assays, we have optimized the DNA amount for linear-DNA transfection. To further optimize the PCR-product based transfection method, we examined whether the expression level of recombinant protein under optimum condition depend on post-transfection incubation time. After transfecting with the linear DNA encoding AtCRY2–GFP, HEK293T cells were then incubated at different time points (8, 16, and 32 h) before the microscopy and immunoblot detection. After 8-h incubation, 3.7% cells expressed the recombinant AtCRY2–GFP, which was analyzed by a fluorescence microscope. As shown in **Figure 2D**, although the cell number increased with longer incubation time, the transfection rate of AtCRY2–GFP approached the plateau (55.5%) after 16hour incubation (**Figure 2E**). The prolonged incubation time increased the number of cells; however, it failed to increase the efficiency of transfection. It implied that HEK293T cell might lose its expression capacity for linear DNA-encoding protein in a long period cultivation. This could be explained by assuming that the linear DNA is not integrated into the genome and can be lost by environmental factors changes and cell division (Rosenberg, 2013). Additionally, the cells expressing the foreign gene usually grow more slowly, which are lost from the population during a long period cultivation (Kim and Eberwine, 2010).

### Analyzing the Photo-Absorption of HEK293T-Expressed Cryptochromes

Cryptochromes sense the blue light in a chromophore-dependent manner, which non-covalently binds to the PHR domain of cryptochromes (Lin et al., 1995). During recombination or purification, FAD was absent or released easily from cryptochromes (Malhotra et al., 1995). For example, the *E. coli*-expressed AtCRY2 is a flavin-deficient protein that is “blind” in blue-light responses. The insect cell was widely used for AtCRY2 expression in previous studies (Liu et al., 2008; Li et al., 2011; Gao et al., 2015), in which the recombinant AtCRY2 absorb blue light via oxidized FAD. However, AtCRY2 purified from insect cell demonstrated partial loss of light responses and constitutive activity in binding AtCIB1 regardless of lighting conditions (Liu et al., 2008).

**FIGURE 3:**
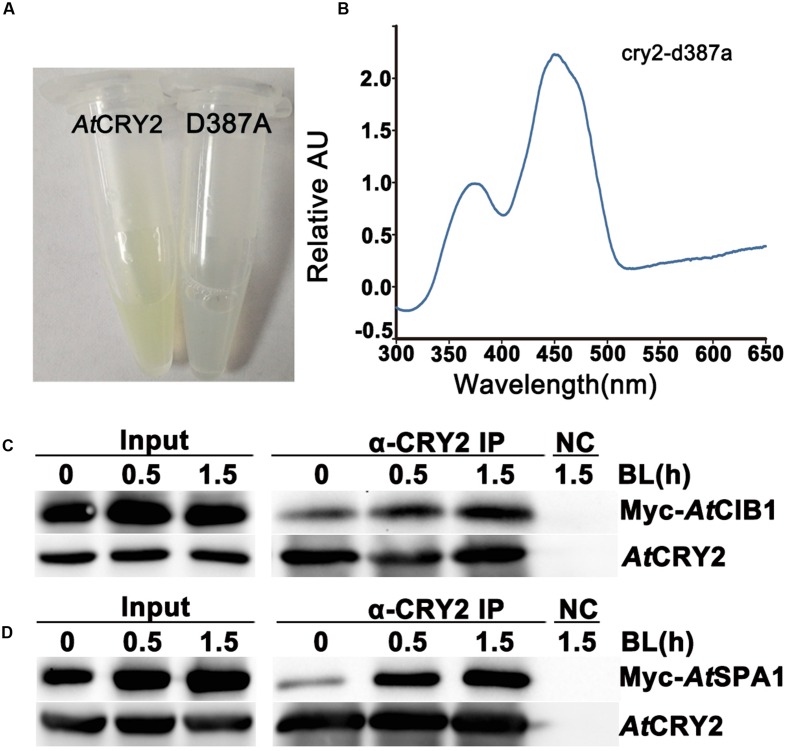
**Biochemical analysis of *Arabidopsis* CRY2 expressed in HEK293T cell.** **(A)** For expression and purification of the recombinant protein, 6xHis-AtCRY2 and 6xHis-AtCRY2^D387A^ were transfected with Calcium Phosphate, respectively. After 24 hours of incubation, cells were collected and lysed. Recombinant AtCRY2 and AtCRY2^D387A^ were purified with Ni-NTA agarose beads. The AtCRY2 exhibited a faint yellow color after enrichment. **(B)** The absorption spectrum of recombinant AtCRY2 and AtCRY2^D387A^ were detected by a full-length spectrophotometer, and the related absorption curve was calculated by the following equation: [absorption of CRY2 - absorption of CRY2^D387A^]. **(C,D)** Co-IP analysis showing blue light dependent interaction of AtCRY2–AtCIB1 **(C)** and AtCRY2–AtSPA1 **(D)** in mammalian cells. The HEK293T cells co-transfected with AtCRY2–AtCIB1 or AtCRY2–AtSPA1 were incubated in dark condition. Cells were irradiated with blue light (50 μmol m^-2^ s^-1^) for indicated time before harvest. Total protein extraction and immunoprecipitation (IP) product prepared by AtCRY2 antibody were first probed with AtCRY2 antibody, stripped, and re-probed with Myc antibody.

To investigate whether the recombinant AtCRY2 function properly in our PCR-product based HEK293T expression system, we next analyzed the absorption spectra of HEK293T-expressed AtCRY2. In order to obtain sufficient amount and qualified recombinant AtCRY2 for spectroscopic analysis, AtCRY2 was fused to 6xHis tag and purified with nickel beads. The AtCRY2^D387A^ mutant protein was also expressed and purified as a negative control. The residue D387 of *Arabidopsis* CRY2 is included in the FAD-binding pocket motif conserved in cryptochromes from plant to human. The AtCRY2^D387A^ mutant protein, in which the aspartic acid is replaced with alanine at position 387, does not contain flavin. In contrast to AtCRY2^D387A^, the concentrated AtCRY2 exhibited a faint yellow color after precipitation (**Figure 3A**). The relative absorption spectra of AtCRY2 expressed in HEK293T have a maximum absorption at 450 nm, which is normalized with the absorption spectra of AtCRY2^D387A^ (**Figure 3B**). The spectra are similar to that of the AtCRY2 expressed from insect cells (Lin et al., 1995; Liu et al., 2008). According to previous studies, AtCRY2 also associates with a second chromophore, MTHF. However, it usually disassociates from the recombinant protein during purification. Our result suggested CRY2 could absorb blue light via FAD, or possibly under the co-effect of FAD and MTHF, to accomplish its photo-activity in HEK293T cells.

### Investigating the Photo-Biochemical Activity of HEK293T-Expressed Cryptochromes

To further analyze the photo activity of HEK293T-expressed AtCRY2, we next examined whether the recombinant AtCRY2 interacts with AtCIB1 or AtSPA1 in a blue light dependent manner. In previous studies, we have proved the accumulation of both AtCRY2–AtCIB1 and AtCRY2–AtSPA1 complexes are stimulated by blue light in plant cells (Liu et al., 2008; Zuo et al., 2011). Since insect-cell-expressed AtCRY2 interacts with AtCIB1 regardless of blue light in vitro, the HEK293T expression system was expected to generate fully photo-responsive AtCRY2 on the study of photo-biochemical mechanism in vitro. In this experiment, AtCRY2 was co-transfected with Myc-AtCIB1 or Myc-AtSPA1 into dark-grown HEK293T cells. Transfected cells were then exposed to blue light (50 μmol m^-2^ s^-1^) for the indicated time and subjected to co-IP analyses. In the sample irradiated with 0.5 and 1.5 h blue light, AtCRY2 was co-precipitated with AtCIB1 and AtSPA1 (**Figures 3C,D**). In contrast, little AtCIB1 or AtSPA1 co-precipitated with AtCRY2 in dark-grown sample. This result suggested HEK293T-expressed AtCRY2 is in possession of full light response property for protein–protein interaction, which is similar to that in plant cells.

**FIGURE 4:**
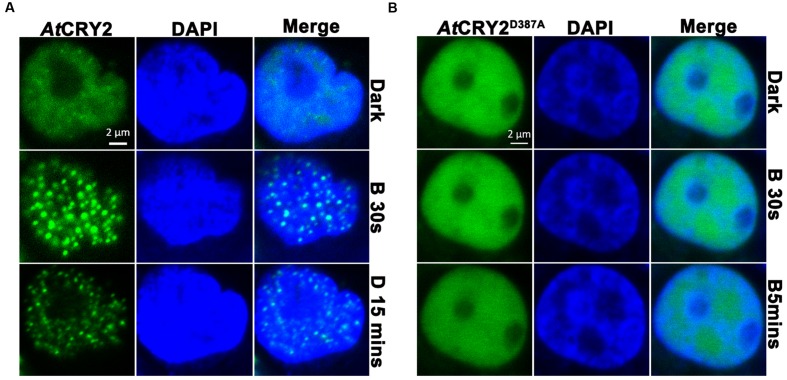
**Photobody formation and observation of AtCRY2–GFP.** Cells were seeded in glass-bottom culture dish (20 mm), 3 μg of linear DNA encoding AtCRY2–GFP or AtCRY2^D387A^–GFP was transfected by Lipofectamine 3000 method. After 24-h incubation, cells were incubated with 500 μl of PBS (pH 7.2) supplemented with 5 μl DAPI (100×) at room temperature for 10 min. Cells were washed twice before observation. The transfected cells were irradiated with 488 nm laser for indicated time, followed by incubation in dark for 15 min and visualized by fluorescence microscopy. **(A)** AtCRY2–GFP. **(B)** AtCRY2^D387A^–GFP. Figures were merged by Zeiss ZEN2 software.

It has been proposed that AtCRY2 majorly function and is regulated in the photobody. After a few seconds of blue light irradiation, AtCRY2 visually forms discernable photobodies in the nucleus, which seems much faster than other photo-activities of AtCRY2 (Yu et al., 2009; Zuo et al., 2012). Recently, it suggested AtCRY2 also could form the light-induced photobody in mammalian cells (Ozkan-Dagliyan et al., 2013). We expanded the investigation to examine the properties of AtCRY2 photobodies expressed by PCR-product-based HEK293T expression system. As shown in **Figure 4**, *AtCRY2* and *AtCRY2*^D387A^ exhibited the uniform localization in nucleus. In contrast with AtCRY2^D387A^, AtCRY2 formed the photobodies in a blue light dependent manner. This result was identical with previous studies, in which AtCRY2 was expressed with other methods or in other species (Yu et al., 2009; Ozkan-Dagliyan et al., 2013).

## Discussion

In the present study, we developed a PCR-product-based transfection method to express plant photoactive protein in HEK293T cell. Our result indicated that the holoprotein of recombinant AtCRY2 expressed with this method is photochemically active and robust, in contrast to other expression systems previously utilized. For example, the *E. coli*-expressed AtCRY2, whose light activity is deficient, usually disassociates chromophore; the insect-cell-expressed AtCRY2, which have partially lost light response, interacts with AtCIB1 regardless of blue light; the low yield of yeast-expressed CRY2 is not sufficient for photo-biochemical assay, etc. In the present method, AtCRY2 could be expressed for the biochemical analyses out of plant cell, e.g., identification of the direct interaction with other proteins, analyses of photobody formation in a non-plant-component circumstance (Taslimi et al., 2016). In addition, this method is simplified from the traditional HEK293T transfection protocol and is an “easy to use” procedure for plant protein expression. For example, the whole transfection process of this method only need 30 min (**Figure 1**), except the maintenance of HEK293T cell cultivation and the preparation of the linear DNA template. Furthermore, this method is also suitable for 96-well plate format and requires no plasmid construction, we then envision two broad uses of this method in the biochemical study of plant proteins: interrogating the plant protein–protein interactions and analyzing the plant protein function.

When HEK293T is used as a tool for querying protein–protein interactions, this method has a number of advantages, especially for large-scale analyses. It requires no plasmid construction, and the linear-DNA containing bait or prey could be prepared with high-throughput overlap PCR. Combined with BiFC or Split-Luc technology, this method could investigate the dynamics of protein–protein interaction in real time under various flux conditions, for example, in the dark to blue light condition. In a six-well-plate expression format, this method could also be applied for Co-IP based protein–protein interaction analyses. Considering the mutation introduced by PCR, it suggested to verify the interaction with plasmid-based recombinant protein after the large-scale screening or to synthesize the linear-DNA directly. In addition to querying the protein–protein interaction, we expect this method to be even more powerful in protein function analyses. For example, analyzing the transcriptional activity of a transcription factor in real time by co-transfecting the transcription factor and the target DNA fused with a report gene. The uncertain DNA target could be even screened by co-transfecting the transcription factor and a set of randomly synthesized DNA mixture.

## Author Contributions

ZZ and CL designed experiments and wrote the manuscript; LY and WX optimized the transfection method and analyzed the biochemical activity of recombinant proteins; WD and WM investigated the formation of photo bodies; JG, QL and QW maintained the cell culture; CZ organized the flow chart.

## Conflict of Interest Statement

The authors declare that the research was conducted in the absence of any commercial or financial relationships that could be construed as a potential conflict of interest.
